# Susceptibility to Scams in Older Black and White Adults

**DOI:** 10.3389/fpsyg.2021.685258

**Published:** 2021-07-12

**Authors:** S. Duke Han, Lisa L. Barnes, Sue Leurgans, Lei Yu, Christopher C. Stewart, Melissa Lamar, Crystal M. Glover, David A. Bennett, Patricia A. Boyle

**Affiliations:** ^1^Department of Family Medicine, University of Southern California, Los Angeles, CA, United States; ^2^Department of Neurology, University of Southern California, Los Angeles, CA, United States; ^3^Department of Psychology, University of Southern California, Los Angeles, CA, United States; ^4^School of Gerontology, University of Southern California, Los Angeles, CA, United States; ^5^Department of Psychiatry and Behavioral Sciences, Rush University Medical Center, Chicago, IL, United States; ^6^Rush Alzheimer's Disease Center, Rush University Medical Center, Chicago, IL, United States; ^7^Department of Neurological Sciences, Rush University Medical Center, Chicago, IL, United States; ^8^Department of Neurology, Indiana University School of Medicine, Indianapolis, IN, United States

**Keywords:** susceptibility to scams, race, disparities, contextual, affective

## Abstract

Previous reports on racial differences in scam susceptibility have yielded mixed findings, and few studies have examined reasons for any observed race differences. Older Black and White participants without dementia (*N* = 592) from the Minority Aging Research Study and the Rush Memory and Aging Project who completed a susceptibility to scam questionnaire and other measures were matched according to age, education, sex, and global cognition using Mahalanobis distance. In adjusted models, older Black adults were less susceptible to scams than older White adults (Beta = −0.2496, SE = 0.0649, *p* = 0.0001). Contextual factors did not mediate and affective factors did not moderate this association. Analyses of specific items revealed Black adults had greater knowledge of scam targeting of older adults and were less likely to pick up the phone for unidentified callers. Older Black adults are less susceptible to scams than demographically-matched older White adults, although the reasons remain unknown.

## Introduction

Adults over the age of 65 are at risk for financial scam and fraud (AARP, 2020). Existing research suggests that loss of wealth due to scams can result in a reduction in independence and other associated negative outcomes (Templeton and Kirkman, 2007; Lichtenberg et al., 2020; Weissberger et al., 2020). The effects are particularly troubling since older age is typically associated with limited employment opportunities and less time to recover from financial losses (Dessin, 2000; Jackson and Hafemeister, 2011). In a hearing before the United States Senate, it was reported that almost half of older adults consider fraud a higher concern than health or terrorism threats (U. S. Senate, 2005), and in 2018, the Senior Safe Act was signed into law with bipartisan support to help combat financial exploitation of older adults (U. S. Senate, 2018). From a health perspective, our group has linked scam susceptibility in old age with poorer cognition and psychological well-being (James et al., 2014), mild cognitive impairment (Han et al., 2016a), incident Alzheimer's Disease (Boyle et al., 2019), brain gray matter density (Han et al., 2016b), and white matter integrity (Lamar et al., 2020). Other groups have done pioneering work in cyberscams, a growing concern. For example, different types of cyberscams appear to be associated with different, and sometimes opposing, victim characteristics (Whitty, 2020), and fraud susceptibility has been linked to certain cognitive and psychological predictors (Jones et al., 2019). However, most existing research has been conducted primarily in older White adults. Given the important implications for health and well-being, a greater understanding of susceptibility to scams in diverse older adult populations is crucial from a public health perspective.

Little is known about racial differences in scam susceptibility, though susceptibility might be inferred from research on actual scam victimization. However, research findings on racial differences in actual scam victimization have been inconsistent. Some work has suggested that older Black adults are more likely to experience financial exploitation than older White adults, while other studies have not found racial differences. For example, rates of financial exploitation were found to be higher for older Black adults in a telephone survey of over 4,000 in New York (Peterson et al., 2014), and in another study of over 900 older adults in Pennsylvania (Beach et al., 2010). However, after controlling for demographics and other factors, race differences in fraud victimization were not observed in a separate Federal Trade Commission report (Federal Trade Commission, 2007). In more recent work on scams from the Financial Industry Regulatory Authority (FINRA), the Better Business Bureau (BBB), and the Stanford Center on Longevity, no differences in engagement with scammers or actual victimization were observed by race in adjusted statistical models of over 1,400 Americans and Canadians who were targeted and reported a scam (Deliema et al., 2019).

Although greater scam victimization may be due to greater vulnerability to scams on the part of older adults, victimization and vulnerability can be dissociable considerations. For example, greater victimization may be due to more aggressive targeting of a particular group by scammers rather than due to greater susceptibility. To our knowledge, no study has yet investigated racial differences in scam susceptibility in older adults. To address this gap in the literature, we investigated whether a racial difference exists in susceptibility to scams in a large, well-characterized group of non-demented older Black adults demographically and cognitively matched to older White adults from two Rush Alzheimer's Disease Center (RADC) cohort studies of aging. Given the mixed literature on racial differences in victimization (Federal Trade Commission, 2007; Beach et al., 2010; Peterson et al., 2014; Deliema et al., 2019) and the dearth of literature on scam susceptibility among Black adults, we hypothesized that as a result of the well-known structural inequities and strain of living in inhospitable environments, older Black adults would be more susceptible to scams than demographically-matched older White adults. It is well-known that social and environmental settings over the life course often vary by race, primarily due to well-documented structural inequalities and systems of oppression (Leyser-Whalen et al., 2011; Bailey et al., 2017), and unequal access to supportive resources (Feagin and Bennefield, 2014). Thus, we were also interested in examining whether differences in contextual factors, such as discrimination, current socioeconomic status, and domain-specific financial and health literacy, would fully account for (mediate) racial differences in susceptibility to scams. Similarly, we investigated affective factors such as trust, risk aversion, and loneliness, all of which have been shown to vary by race (Rosen et al., 2003; Moreno-John et al., 2004; Han et al., 2017), to determine whether these may impact the strength of (moderate) any observed racial differences. Finally, in order to better understand whether specific aspects of scam susceptibility may be driving any perceived racial differences, we investigated racial differences in each item on the susceptibility to scams scale. As in previous related work in these cohorts (e.g., Han et al., 2020), Black and White participants were matched according to age, education, sex, and global cognition using a robust statistical methodology (Mahalanobis Distance).

## Materials and Methods

### Participants

Older Black participants of the Rush Alzheimer's Disease Center (RADC) Minority Aging Research Study (MARS; Barnes et al., 2012) and the Rush Memory and Aging Project (MAP; Bennett et al., 2018) completed a decision making substudy which included a susceptibility to scams measure. MARS and MAP are large longitudinal cohort studies of aging based in the greater Chicago region and are harmonized in data collection and data management approaches. This harmonization facilitates data pooling across studies. The RADC decision making substudy began in 2010 in MAP and in 2017 in MARS. Among 775 older Black participants in MARS, 306 enrolled in the decision making substudy and completed decision making measures and a clinical evaluation. Eight were excluded due to a dementia diagnosis, and 2 were excluded due to missing data on key variables of interest, leaving 296 Black participants. Next, we identified White participants with decision making data. There were 1,187 White participants who could serve as potential matches for Black participants. Among those, 62 had dementia, and 7 had missing data on variables of interest, leaving 1,118 potential White participant matches.

Mahalanobis Distance matching was used to identify an equal number of White participants (*N* = 296) to Black participants (*N* = 296) according to the pre-selected variables of age, education, sex, and global cognition for this study. Age (calculated from birthdate to date of decision making assessment), sex (male coded as 1 and female coded as 0), and education (self-reported number of years completed) were included as matching variables since these have previously demonstrated associations with susceptibility to scams (James et al., 2014). Global cognition was included as a matching variable (in addition to demographics) as it has been shown to be associated with susceptibility to scams (James et al., 2014) and because there are well-documented racial differences in level of cognitive performance in old age (Weuve et al., 2018), including for Black and White adults in the current research cohorts (Wilson et al., 2015b). Age was matched according to four categories: ≥60 years old to <70 years old, ≥70 years old to <80 years old, ≥to 80 years old to 90 years old, and ≥90 years old to <100 years old. Education was matched according to three categories: from 0 to 12 years of education, 13 to 16 years of education, and >16 years of education. Global cognition was matched within a range of ± 0.25 *z*-score at the individual level. This Mahalanobis Distance matching approach has been utilized in previous work by our group (Han et al., 2020).

### Race

Race was determined by self-report in response to the question, “With which group do you most closely identify yourself?” Participants could respond according to 1990 U.S. Census race categories, which included categories of “White” and “Black or African American.”

### Cognition

An established cognitive battery including 18 measures was utilized for assessment of global cognition (Wilson et al., 2003, 2015a; Bennett et al., 2018). Measures in the battery assessed a wide array of cognitive abilities. The battery included the oral version of the Symbol Digit Modalities Test, Verbal Fluency, Boston Naming, Word List Memory; Word List Recall and Word List Recognition from the procedures established by the CERAD; immediate and delayed recall of Logical Memory Story A; immediate and delayed recall of the East Boston Story; Judgment of Line Orientation, Standard Progressive Matrices, Number Comparison, Stroop Color Naming, Stroop Word Reading, Digit Span subtests forward and backward of the Wechsler Memory Scale-Revised, and Digit Ordering. Performance scores on each measure were *z*-score transformed according to the mean and standard deviation of the baseline cognitive assessment of the sample of the parent study in keeping with previous work (Wilson et al., 2015b). Global cognition was calculated by averaging the *z*-scores across all tests. Standardized criteria for dementia diagnosis were utilized (McKhann et al., 1984).

### Susceptibility to Scams

The susceptibility to scams scale is a five-item self-report measure in which participants rated their agreement to a statement according to a 7-point Likert scale (strongly agree to strongly disagree). The five statements included in the measure have been previously published [specific items published in James et al. (2014)] and address topics such as telemarketing behaviors, older adults being targeted by con-artists, and suspiciousness of claims that seem too good to be true. The measure is based on findings from the AARP and the Financial Industry Regulatory Authority (FINRA) Risk Meter, a measure of poor and risky financial decision making that is utilized in finance studies (AARP, 1999; Financial Industry Regulatory Authority, 2013). Participants are asked to rate their level of agreement with each question using a Likert scale ranging from 1 to 7 (1 = strongly agree, 2 = agree, 3 = slightly agree, 4 = neither agree or disagree, 5 = slightly disagree, 6 = disagree, 7 = strongly disagree). The total score for susceptibility to scams was calculated by averaging the five items (with items 1, 2, and 5 reverse coded) so that higher scores correspond to more susceptibility. Performance on this measure is associated with factors related to victimization, such as higher age, lower literacy, lower cognitive function, and lower psychological well-being (James et al., 2014). This measure has also been associated with cognitive decline in older adults without cognitive impairment (Boyle et al., 2012), as well as mild cognitive impairment (Han et al., 2016a), incident Alzheimer's Dementia (Boyle et al., 2019), and neuroimaging structural markers (Han et al., 2016b, Lamar et al., 2020).

### Contextual Factors

#### Self-Reported Discrimination

Self-reported experiences of discrimination were assessed using the Detroit Area Study Everyday Discrimination scale that asked participants to indicate how frequently they experience mistreatment in everyday life without reference to age, race, or any other social status characteristic (Williams et al., 1997). Examples of individual items include “You are treated with less respect than other people,” “You are treated with less courtesy than other people,” and “People act as if they are better than you are.” Frequency for each item is rated on a four-point scale (“often,” “sometimes,” “rarely,” and “never”), and for analyses responses were dichotomized such that items with ratings of “often” or “sometimes” were coded as 1 and items rated as rarely or never were coded as 0. The range for this measure is 0–9; higher scores indicating greater discrimination. This scale has been used in numerous studies of older Black adults and was found to have good internal consistency and validity and found to be related to health outcomes (Barnes et al., 2004; Lewis et al., 2010).

#### Socioeconomic Status

Socioeconomic status was rated using a show-card methodology as previously described (Bennett et al., 2018). Self-reported annual income was ranked according to 10 possible categories: 1: $0–$4999, 2: $5000–$9999, 3: $10,000–$14,999, 4: $15,000–$19,999, 5: $20,000–$24,999, 6: $25,000–$29,999, 7: $30,000–$34,999, 8: $35,000–$49,999, 9: $50,000–$74,999, 10: >$75,000.

#### Financial and Health Literacy

Financial and health literacy was measured with 32 questions that evaluate knowledge of financial and health information and concepts (Bennett et al., 2012; James et al., 2012; Boyle et al., 2013). There were 23 questions on financial literacy, many of which were modified from questions used on the Health and Retirement Survey (Lusardi and Mitchell, 2007). Questions assessed the ability to perform calculations (numeracy), as well as knowledge of financial concepts and entities such as stocks, bonds, and compound interest. There were nine questions on health literacy, which included questions on Medicare and Medicare Part D, following prescription instructions, leading causes of death in older persons, and understanding drug risks. Because of the difference in number of questions across the domains of literacy, health and financial literacy scores were expressed as the percent correct (from 0 to 100) out of total items within each domain, and total literacy was the mean of these two percentages. The test–retest reliability for the total literacy score over a 1-year interval was adequate (Intraclass Correlation Coefficient = 0.75; Lohr, 2002). We have previously shown that this measure of literacy is related to engagement in health promoting behaviors, functional status, aspects of physical and mental health, financial and healthcare decision making, and cognitive decline (Bennett et al., 2012; James et al., 2012; Boyle et al., 2013). We also found that this measure mediates racial differences in financial and healthcare decision making among older Black and White adults (Han et al., 2020).

### Affective Factors

#### Trust

Trust was measured using eight self-report questions from the NEO Personality Inventory (Costa and McCrae, 1992). The range of scores is 0–32 and higher scores indicate an increased level of trust. Three of the eight items are flipped (reverse-scored), and items ask the respondent to indicate their level of trust or suspicion of other people.

#### Risk Aversion

Risk aversion was assessed with 10 questions used in standard behavioral economics approaches. An example of one of these questions is “Would you prefer $15 for sure, OR a coin toss in which you will get $[an amount > $15] if you flip heads or nothing if you flip tails?” Possible gains ranged from $21.79 to $151.19 and gain amounts were varied across questions. Any gamble that offered a potential gain of $30 resulted in the same long run average or expected utility, and any gamble that was over $30 resulted in a greater than expected utility. Therefore, any gamble over $30 was a “preferred” choice. This measure has been associated with cognition (Boyle et al., 2011), cognitive decline (James et al., 2015), resting-state brain networks (Han et al., 2012), and Alzheimer's dementia (Wilson et al., 2019) in older adults. Higher values indicated greater risk aversion.

#### Loneliness

Loneliness was assessed with a modified version of the de Jong-Gierveld Loneliness Scale (de Jong-Gierveld and Kamphuis, 1985; de Jong-Gierveld, 1987). The following 5 items were examined: “I experience a general sense of emptiness,” “I miss having people around,” “I feel like I don't have enough friends,” “I often feel abandoned,” and “I miss having a really good friend.” An average of the item scores yielded a total score that ranged from 1 to 5, with higher values indicating greater loneliness. This measure has been associated with higher risk of Alzheimer's dementia (Wilson et al., 2007), and has been observed to be lower in older Black adults with HIV vs. older White adults with HIV (Han et al., 2017).

### Statistical Analysis

Descriptive and bivariate statistics characterized non-demented older Black and older White adults. Chi-square tests were used for categorical variables, *t*-tests were used for continuous variables, and non-parametric Wilcoxon Rank Sum tests were reported if distributions were skewed. To determine whether there was a racial difference in susceptibility to scams, linear regression models were performed to examine the associations between race (Black = 1, White = 0) and susceptibility to scams. Despite the matching, all models included terms to control for the potentially confounding effects of age, education, sex, and global cognition. As described above, because of well-known variations by race in social and environmental settings over the life course, we were also interested in examining whether differences in the contextual factors of discrimination, current socioeconomic status, and domain-specific financial and health literacy would fully account for (mediate) racial differences in susceptibility to scams. Similarly, we were further interested in whether racial differences differ by the affective factors of trust, risk aversion, and loneliness. To address these, linear regression models were performed to examine the potential mediating effects of contextual factors (i.e., self-reported discrimination, socioeconomic status, financial and health literacy) and the potential moderating effects of affective factors (i.e., trust, risk aversion, and loneliness). Finally, in order to better understand what specific aspects of scam susceptibility may be driving any perceived racial differences, we investigated racial differences in each item on the susceptibility to scams scale. Analyses were programmed in SAS version 9.4 software.

## Results

### Descriptive Data

As expected with Mahalanobis Distance matching, no differences between races were observed for age, education, sex, and global cognition. The sample was predominantly female (each group had 53 males and 243 females) and had a mean post-high school level of education. Differences according to race were observed in susceptibility to scams; the contextual factors of self-reported discrimination, socioeconomic status, and financial and health literacy; and the affective factors of trust, risk aversion, and loneliness. Older Black adults showed less susceptibility to scams, greater self-reported discrimination, lower socioeconomic status, lower financial and health literacy, lower trust, greater risk aversion, and less loneliness than older White adults. See Table 1.

**Table 1:** Demographic, cognitive, and other descriptive data.

	**Black (*****N*** **=** **296)**		**White (*****N*** **=** **296)**			
	**Mean**	**SD**	**Mean**	**SD**	***t, Z***	***p***
Age	77.903	6.614	78.190	6.703	*t* = 0.52	0.6014
Education	15.128	3.236	15.149	3.054	*Z* = 0.3932	0.6942
Global cognition	0.061	0.541	0.120	0.511	*Z* = 1.2219	0.2217
Susceptibility to scams	2.386	0.865	2.616	0.797	*Z* = 4.0428	<0.0001
Self-reported discrimination	1.881	2.152	0.739	1.257	*Z* = 6.9496	<0.0001
Socioeconomic status	6.144	2.700	6.981	2.450	*Z* = 3.6025	0.0003
Financial and health literacy	60.608	12.825	67.918	13.055	*t* = 6.8600	<0.0001
Trust	20.836	4.051	24.054	3.465	*Z* = 8.6692	<0.0001
Risk aversion	0.438	0.313	0.292	0.299	*Z* = −8.0460	<0.0001
Loneliness	2.022	0.631	2.160	0.607	*Z* = −2.6904	0.0071

*Age and education are presented in years. Global cognition is a mean of z-scores. Susceptibility to scams is total score. For age and financial and health literacy, t-values are reported. For education, global cognition, susceptibility to scams, self-reported discrimination, socioeconomic status, trust, risk aversion, and loneliness, Wilcoxon Z-values are reported*.

### Susceptibility to Scams

In linear regression models controlling for demographic variables (age, education, and sex), age and education were independently associated with susceptibility to scams (Table 2, Model 1). Higher age and fewer years of education were associated with greater scam susceptibility. Global cognition also was independently associated with susceptibility to scams such that better cognitive performance was associated with less scam susceptibility (Table 2, Model 2). Furthermore, in models adjusted for age, education, sex, and global cognition, older Black adults showed less susceptibility to scams than White adults (Table 2, Model 3). Using age as a frame of reference, older Black adults responded in a manner consistent with being 16.1 years younger than older White adults on the susceptibility to scams measure.

**Table 2:** Association of race with susceptibility to scams.

	**Model 1**	**Model 2**	**Model 3**
**Estimate (standard error**, ***p*****-value)**			
Age	0.0263 (0.0051, <0.0001)	0.0164 (0.0053, 0.0020)	0.0155 (0.0052, 0.0031)
Sex (male = 1, female = 0)	0.1462 (0.0877, 0.0959)	0.0901 (0.0865, 0.2976)	0.0878 (0.0855, 0.3045)
Education	−0.0363 (0.0107, 0.0007)	−0.0158 (0.0112, 0.1560)	−0.0149 (0.0110, 0.1783)
Global cognition		−0.3698 (0.0705, <0.0001)	−0.3891 (0.0699, <0.0001)
Race (Black = 1, White = 0)			−0.2496 (0.0649, 0.0001)

*Dependent variable is susceptibility to scams total score. For sex, male is coded as 1 and female is coded as 0. For race, Black is coded as 1 and White is coded as 0*.

### Contextual and Affective Factors

In separate models examining contextual factors (i.e., self-reported discrimination, socioeconomic status, financial and health literacy), socioeconomic status (Supplementary Table 2) and financial and health literacy (Supplementary Table 3) were associated with susceptibility to scams in all participants. However, no contextual factor mediated the racial difference in susceptibility to scams (Supplementary Tables 1–3). In separate individual models considering affective factors (i.e., trust, risk aversion, and loneliness), only loneliness was associated with susceptibility to scams (Supplementary Table 6). No affective factor moderated the racial difference in susceptibility to scams (Supplementary Tables 4–6). We also explored whether considering all factors together had an impact on racial differences. When contextual factors were considered together (Supplementary Table 7, Model 1) and affective factors were considered together (Supplementary Table 7, Model 2), the race difference was still significant. Finally, when all contextual and affective factors were considered together (Supplementary Table 7, Model 3), the race difference remained significant.

### Susceptibility to Scams: Analysis of Individual Items

To better understand the potential driving factors for the racial difference observed in the susceptibility to scams summary measure, we examined racial differences in response to individual items on the measure in *post-hoc* Wilcoxon analyses (Table 3). Older Black adults differed from older White adults on two of the five items: (Item 1, flipped scoring statement) “I feel I have to answer the phone whenever it rings, even if I do not know who is calling,” and (Item 4, non-flipped scoring statement) “Persons over the age of 65 are often targeted by con-artists.” Specifically, older Black adults overall were less inclined to answer the phone whenever it rings, and agreed more with the statement that persons over the age of 65 are often targeted by scammers compared to older Whites.

**Table 3:** Susceptibility to scams item response by race.

	**Black (*****N*** **=** **296)**		**White (*****N*** **=** **296)**			
	**Mean**	**SD**	**Mean**	**SD**	***Z***	***p***
Item 1: I feel I have to answer the phone whenever it rings, even if I do not know who is calling.	3.490	2.055	4.676	2.062	6.8043	<0.0001
Item 2: I have difficulty ending a phone call, even if the caller is a telemarketer, someone I do not know, or someone I did not wish to call me.	2.135	1.455	2.182	1.438	0.5724	0.5670
Item 3: If something sounds too good to be true, it usually is.	2.000	1.083	1.882	0.885	−1.0539	0.2919
Item 4: Persons over the age of 65 are often targeted by con-artists.	1.747	0.689	1.909	0.695	3.3736	0.0007
Item 5: If a telemarketer calls me, I usually listen to what they have to say.	2.557	1.672	2.432	1.512	−0.3073	0.7586

*Wilcoxon Z-values are reported. Items 1, 2, and 5 are flipped scoring statements, meaning that higher scores indicate higher agreement. Items 3 and 4 are non-flipped scoring statements, meaning that lower scores indicate higher agreement*.

## Discussion

In a group of more than 500 older Black and White adults demographically and cognitively matched using a robust statistical approach, we found that older Black adults showed less susceptibility to scams than older White adults. The contextual factors of self-reported discrimination, socioeconomic status, and financial and health literacy did not mediate this difference, and the affective factors of trust, risk aversion, and loneliness did not moderate this difference. In *post-hoc* analyses of the individual questions comprising susceptibility to scams, older Black adults indicated less willingness to pick up the phone when the caller was unknown, and were more likely to endorse the notion that older adults often are targeted by con-artists.

These findings provide greater clarity on the issue of racial differences in susceptibility to scams. Given previous work on financial exploitation (e.g., Beach et al., 2010; Peterson et al., 2014), one might have predicted that older Black adults would show greater susceptibility to scams given greater reported rates of victimization. However, while older adults in general are targeted more for scams and fraud (AARP, 2020), it has been reported that Black and Latino communities in particular may experience disproportionately more scam *targeting* by perpetrators than White communities (Federal Trade Commission, 2016). Because of disproportionately higher *targeting* of diverse populations by scammers, this higher targeting may manifest as higher *victimization* rates. However, *susceptibility* may be lower for a number of reasons, including effective public awareness campaigns of risks, increased vigilance because of societal racial bias (Carter et al., 2013), and expectations of racism (Lewis et al., 2019). It is reasonable to assume that vigilance may be one mechanism whereby a particular community might show less scam susceptibility. The differences in item responses seem to support this line of reasoning in our study, and there is growing evidence to support that Black communities are highly vigilant about potential negative social outcomes (Himmelstein et al., 2015; Lewis et al., 2019).

Contrary to our hypotheses, we did not observe that particular contextual factors such as discrimination, socioeconomic status, and financial and health literacy mediate racial differences, nor that particular affective factors such as trust, risk aversion, and loneliness moderate racial differences. Given the importance of discrimination as an explanatory variable for racial differences, it is not clear why self-reported discrimination did not mediate racial differences in scam susceptibility. The everyday discrimination scale used in the current study measures chronic instances of unfair treatment and microaggressions (Williams et al., 1997). However, experiences of discrimination are multifactorial, and it is possible that specific and more personally significant discriminatory events may have mediated racial differences in scam susceptibility. It is also noteworthy that reported levels of discrimination were low across the entire sample, and this may also explain why they did not mediate racial differences.

Perhaps other factors may play a role in racial differences in scam susceptibility. One potential factor not measured in the current study that might account for the racial difference is level of exposure to others who have experienced scams. For example, older Black adults may have peers who experienced scams or were defrauded. This exposure to others' victimization might result in greater awareness to potential scam situations. Secondly, although our measure of interpersonal trust did not moderate racial differences, it is possible that institutional trust might have had a moderating effect given the well-documented evidence of medical mistrust among older Black adults due to multiple examples of past abuses in medical practice and medical research, including, but not limited to, forced enrollment without consent, subjugation to dubious experimental procedures, purposeful exposure to toxins, farming for bodily fluids or tissue, involuntary sterilization, and intentional withholding of health-improving treatments (Moreno-John et al., 2004; Washington, 2008; Scharff et al., 2010; Alsan and Wanamaker, 2018; Webb Hooper et al., 2019). Lastly, a measure of lifetime scam targeting might have also been helpful to incorporate. Each of these factors may be directions for future research.

Limitations of the present study need to be acknowledged. The cross-sectional nature of the study precludes any causal inferences. Our susceptibility to scams measure includes some items that may pertain to phone scams in particular. The ability to focus on different types of scam and fraud is limited with our measure. Participants were selected, and although we used a robust matching strategy to minimize demographic differences between Black and White participants, it is possible that selection bias exists that is driving the observed racial differences, and this could limit generalizability of findings. In particular, the older Black adults in our sample likely represent a more resilient group with higher education and better health than older Black adults of this age from the general population, with those less susceptible to scams surviving in greater numbers into older age. However, the fact that we find racial differences in a sample matched on age and education suggests that racial differences may be even larger in the general population. Future population-based studies of Black adults with a wider distribution of age and education are needed to replicate these findings. Furthermore, our study includes mostly women and persons from an urban city in the Midwest and does not fully represent the broader diversity of older adults in terms of social-demographic and other population-based factors. It is currently unknown how predictive the susceptibility to scams measure is of future scam victimization. Efforts are ongoing to establish this. Finally, as mentioned above, there may be other contextual and affective factors that may explain some of the variation between groups that we have not yet considered or were not able to consider presently.

This study also has notable strengths. These include a large, well-characterized sample of participants, the use of a robust approach (Mahalanobis Distance) to statistically match participants according to the same levels of age and education, the consideration of contextual and affective factors that differ by race, and the use of a measure of susceptibility to scams that has been robustly associated with significant cognitive and health outcomes in old age. Our study suggests older Black adults are less susceptible to scams than older White adults. Since older Black adults appear to be targeted disproportionately more than older White adults, this finding is encouraging and may indirectly suggest that public awareness campaigns about scams and fraud may be effective. Future work is needed to address the increased scam targeting of diverse older adult communities.

## Data Availability Statement

The datasets presented in this study can be requested at RADC Research Resource Sharing Hub: https://www.radc.rush.edu/.

## Ethics Statement

The studies involving human participants were reviewed and approved by IRB of USC and Rush University. The patients/participants provided their written informed consent to participate in this study.

## Author Contributions

SDH planned the study, supervised the data analysis, and wrote the paper. SL and LY performed all statistical analyses and contributed to revising the paper. LB, CS, ML, CG, DB, and PB helped to plan the study and to revise the manuscript. All authors contributed to the article and approved the submitted version.

## Conflict of Interest

The authors declare that the research was conducted in the absence of any commercial or financial relationships that could be construed as a potential conflict of interest.
