# Supplementary material for: Susceptibility to Scams in Older Black and White Adults
Source: Front Psychol. 2021 Jul 12;12:685258. doi: 10.3389/fpsyg.2021.685258 (PMC8311557; doi:10.3389/fpsyg.2021.685258)
Supplement: Supplementary file 1 [file Table_1.DOCX]

**SUPPLEMENTARY MATERIALS**

**Table S1. Associations of Race and Self-Reported Discrimination with Susceptibility to Scams**

|  | Model 1 | Model 2 | Model 3 | Model 4 | Model 5 |
| --- | --- | --- | --- | --- | --- |
|  | Estimate (Standard Error, *p*-value) | | | | |
| Age | 0.0263 (0.0051, <0.0001) | 0.0164 (0.0053, 0.0020) | 0.0155 (0.0052, 0.0031) | 0.0159 (0.0054, 0.0030) | 0.0156 (0.0053, 0.0033) |
| Sex (Male=1, Female=0) | 0.1462 (0.0877, 0.0959) | 0.0901 (0.0865, 0.2976) | 0.0878 (0.0855, 0.3045) | 0.1005 (0.0873, 0.2503) | 0.0980 (0.0864, 0.2567) |
| Education | -0.0363 (0.0107, 0.0007) | -0.0158 (0.0112, 0.1560) | -0.0149 (0.0110, 0.1783) | -0.0167 (0.0112, 0.1371) | -0.0148 (0.0111, 0.1822) |
| Global Cognition |  | -0.3698 (0.0705, <0.0001) | -0.3891 (0.0699, <0.0001) | -0.3796 (0.0714, <0.0001) | -0.3900 (0.0706, <0.0001) |
| Race (Black=1, White=0) |  |  | -0.2496 (0.0649, 0.0001) |  | -0.2545 (0.0685, 0.0002) |
| Self-Reported Discrimination |  |  |  | -0.0171 (0.0180, 0.3439) | 0.0042 (0.0187, 0.8239) |

**Table S2. Associations of Race and Socioeconomic Status with Susceptibility to Scams**

|  | Model 1 | Model 2 | Model 3 | Model 4 | Model 5 |
| --- | --- | --- | --- | --- | --- |
|  | Estimate (Standard Error, *p*-value) | | | | |
| Age | 0.0263 (0.0051, <0.0001) | 0.0164 (0.0053, 0.0020) | 0.0155 (0.0052, 0.0031) | 0.0171 (0.0057, 0.0028) | 0.0170 (0.0056, 0.0026) |
| Sex (Male=1, Female=0) | 0.1462 (0.0877, 0.0959) | 0.0901 (0.0865, 0.2976) | 0.0878 (0.0855, 0.3045) | 0.1497 (0.0913, 0.1018) | 0.1612 (0.0901, 0.0741) |
| Education | -0.0363 (0.0107, 0.0007) | -0.0158 (0.0112, 0.1560) | -0.0149 (0.0110, 0.1783) | -0.0037 (0.0120, 0.7564) | 0.0004 (0.0119, 0.9764) |
| Global Cognition |  | -0.3698 (0.0705, <0.0001) | -0.3891 (0.0699, <0.0001) | -0.3381 (0.0761, <0.0001) | -0.3516 (0.0751, <0.0001) |
| Race (Black=1, White=0) |  |  | -0.2496 (0.0649, 0.0001) |  | -0.2786 (0.0686, <0.0001) |
| Socioeconomic Status |  |  |  | -0.0422 (0.0147, 0.0044) | -0.0522 (0.0147, 0.0004) |

**Table S3. Associations of Race and Financial and Health Literacy with Susceptibility to Scams**

|  | Model 1 | Model 2 | Model 3 | Model 4 | Model 5 |
| --- | --- | --- | --- | --- | --- |
|  | Estimate (Standard Error, *p*-value) | | | | |
| Age | 0.0263 (0.0051, <0.0001) | 0.0164 (0.0053, 0.0020) | 0.0155 (0.0052, 0.0031) | 0.0152 (0.0053, 0.0043) | 0.0132 (0.0052, 0.0109) |
| Sex (Male=1, Female=0) | 0.1462 (0.0877, 0.0959) | 0.0901 (0.0865, 0.2976) | 0.0878 (0.0855, 0.3045) | 0.1417 (0.0869, 0.1037) | 0.1607 (0.0850, 0.0593) |
| Education | -0.0363 (0.0107, 0.0007) | -0.0158 (0.0112, 0.1560) | -0.0149 (0.0110, 0.1783) | -0.0056 (0.0114, 0.6217) | 0.0004 (0.0112, 0.9724) |
| Global Cognition |  | -0.3698 (0.0705, <0.0001) | -0.3891 (0.0699, <0.0001) | -0.2425 (0.0785, 0.0021) | -0.2153 (0.0768, 0.0053) |
| Race (Black=1, White=0) |  |  | -0.2496 (0.0649, 0.0001) |  | -0.3571 (0.0668, <0.0001) |
| Financial and Health Literacy |  |  |  | -0.0111 (0.0030, 0.0002) | -0.0160 (0.0031, <0.0001) |

**Table S4. Associations of Race and Trust with Susceptibility to Scams**

|  | Model 1 | Model 2 | Model 3 | Model 4 | Model 5 | Model 6 |
| --- | --- | --- | --- | --- | --- | --- |
|  | Estimate (Standard Error, *p*-value) | | | | | |
| Age | 0.0263 (0.0051, <0.0001) | 0.0164 (0.0053, 0.0020) | 0.0155 (0.0052, 0.0031) | 0.0144 (0.0057, 0.0126) | 0.0140 (0.0056, 0.0135) | 0.0139 (0.0056, 0.0139) |
| Sex (Male=1, Female=0) | 0.1462 (0.0877, 0.0959) | 0.0901 (0.0865, 0.2976) | 0.0878 (0.0855, 0.3045) | 0.1312 (0.0934, 0.1611) | 0.1139 (0.0918, 0.2152) | 0.1116 (0.0920, 0.2259) |
| Education | -0.0363 (0.0107, 0.0007) | -0.0158 (0.0112, 0.1560) | -0.0149 (0.0110, 0.1783) | -0.0165 (0.0122, 0.1770) | -0.0113 (0.0120, 0.3481) | -0.0116 (0.0121, 0.3368) |
| Global Cognition |  | -0.3698 (0.0705, <0.0001) | -0.3891 (0.0699, <0.0001) | -0.3642 (0.0783, <0.0001) | -0.3634 (0.0768, <0.0001) | -0.3646 (0.0770, <0.0001) |
| Race (Black=1, White=0) |  |  | -0.2496 (0.0649, 0.0001) |  | -0.3395 (0.0754,  <0.0001) | -0.3452 (0.0768, <0.0001) |
| Trust |  |  |  | 0.0144 (0.0090, 0.1097) | -0.0028 (0.0096, 0.7693) | -0.0073 (0.0147, 0.6209) |
| Race x Trust |  |  |  |  |  | 0.0075 (0.0188, 0.6891) |

**Table S5. Associations of Race and Risk Aversion with Susceptibility to Scams**

|  | Model 1 | Model 2 | Model 3 | Model 4 | Model 5 | Model 6 |
| --- | --- | --- | --- | --- | --- | --- |
|  | Estimate (Standard Error, *p*-value) | | | | | |
| Age | 0.0263 (0.0051, <0.0001) | 0.0164 (0.0053, 0.0020) | 0.0155 (0.0052, 0.0031) | 0.0168 (0.0053, 0.0015) | 0.0158 (0.0052,0.00264) | 0.0158 (0.0052,0.00264) |
| Sex (Male=1, Female=0) | 0.1462 (0.0877, 0.0959) | 0.0901 (0.0865, 0.2976) | 0.0878 (0.0855, 0.3045) | 0.0740 (0.0866, 0.3931) | 0.0783 (0.0858, 0.3615) | 0.0785 (0.0859, 0.3611) |
| Education | -0.0363 (0.0107, 0.0007) | -0.0158 (0.0112, 0.1560) | -0.0149 (0.0110, 0.1783) | -0.0184 (0.0112, 0.1002) | -0.0165 (0.0111, 0.1383) | -0.0164 (0.0111, 0.1416) |
| Global Cognition |  | -0.3698 (0.0705, <0.0001) | -0.3891 (0.0699, <0.0001) | -0.3891 (0.0710, <0.0001) | -0.3992 (0.0704, <0.0001) | -0.3994 (0.0705, <0.0001) |
| Race (Black=1, White=0) |  |  | -0.2496 (0.0649, 0.0001) |  | -0.2311 (0.0667, 0.0006) | -0.2510 (0.1018, 0.0140) |
| Risk Aversion |  |  |  | -0.2181 (0.1069, 0.0418) | -0.1307 (0.1089,  0.2306) | -0.1592 (0.1550, 0.3047) |
| Race x Risk Aversion |  |  |  |  |  | 0.0549 (0.2121, 0.7959) |

**Table S6. Associations of Race and Loneliness with Susceptibility to Scams**

|  | Model 1 | Model 2 | Model 3 | Model 4 | Model 5 | Model 6 |
| --- | --- | --- | --- | --- | --- | --- |
|  | Estimate (Standard Error, *p*-value) | | | | | |
| Age | 0.0263 (0.0051, <0.0001) | 0.0164 (0.0053, 0.0020) | 0.0155 (0.0052, 0.0031) | 0.0144 (0.0054, 0.0078) | 0.0138 (0.0053, 0.0100) | 0.0137 (0.0053, 0.0105) |
| Sex (Male=1, Female=0) | 0.1462 (0.0877, 0.0959) | 0.0901 (0.0865, 0.2976) | 0.0878 (0.0855, 0.3045) | 0.1024 (0.0882, 0.2461) | 0.0994 (0.0873, 0.2553) | 0.0983 (0.0873, 0.2606) |
| Education | -0.0363 (0.0107, 0.0007) | -0.0158 (0.0112, 0.1560) | -0.0149 (0.0110, 0.1783) | -0.0127 (0.0113, 0.2582) | -0.0122 (0.0111, 0.2758) | -0.0123 (0.0111, 0.2687) |
| Global Cognition |  | -0.3698 (0.0705, <0.0001) | -0.3891 (0.0699, <0.0001) | -0.3462 (0.0723, <0.0001) | -0.3654 (0.0718, <0.0001) | -0.3656 (0.0718, <0.0001) |
| Race (Black=1, White=0) |  |  | -0.2496 (0.0649, 0.0001) |  | -0.2377 (0.0660, 0.0003) | -0.2486 (0.0667, 0.0002) |
| Loneliness |  |  |  | 0.1395 (0.0555, 0.0123) | 0.1163 (0.0553, 0.0361) | 0.0564 (0.0774, 0.4666) |
| Race x Loneliness |  |  |  |  |  | 0.1171 (0.1058, 0.2692) |

**Table S7. Associations of Race, Contextual Factors, and Affective Factors with Susceptibility to Scams**

|  | Model 1 | Model 2 | Model 3 |
| --- | --- | --- | --- |
|  | Estimate (Standard Error, *p*-value) | | |
| Age | 0.0141  (0.0056,  0.0117) | 0.0125  (0.0058,  0.0306) | 0.0120  (0.0061,  0.0515) |
| Sex (Male=1, Female=0) | 0.2478  (0.0903,  0.0063) | 0.1129  (0.0939,  0.2299) | 0.2629  (0.0994,  0.0085) |
| Education | 0.0132  (0.0120,  0.2693) | -0.0112  (0.0122,  0.3578) | 0.0101  (0.0130,  0.4389) |
| Global Cognition | -0.1839  (0.0815,  0.0245) | -0.3593  (0.0790,  <0.0001) | -0.1643  (0.0903,  0.0696) |
| Race (Black=1, White=0) | -0.3843  (0.0734,  <0.0001) | -0.3028  (0.0787,  0.0001) | -0.4072  (0.0854,  <0.0001) |
| Self-Reported Discrimination | 0.0020  (0.0187,  0.9169) |  | -0.0006  (0.0215,  0.9793) |
| Socioeconomic Status | -0.0439  (0.0146,  0.0027) |  | -0.0362  (0.0160,  0.0244) |
| Financial and Health Literacy | -0.0161  (0.0032,  <0.0001) |  | -0.0166  (0.0034,  <0.0001) |
| Trust |  | 0.0001  (0.0099,  0.9922) | 0.0072  (0.0103,  0.4838) |
| Risk Aversion |  | -0.1064  (0.1183,  0.3689) | -0.1713  (0.1246,  0.1699) |
| Loneliness |  | 0.0943  (0.0616,  0.1266) | 0.0446  (0.0650,  0.4930) |

**Table S8. Chronbach Alpha Values for Measures**

| **Measure** | **Chronbach Alpha** |
| --- | --- |
| Self-Reported Discrimination | 0.782 |
| Socioeconomic Status | * |
| Financial and Health Literacy | 0.703 |
| Trust | 0.773 |
| Risk Aversion | 0.903 |
| Loneliness | 0.792 |

*Note: Socioeconomic Status was measured with a single question. Because of this, Chronbach Alpha was not able to be computed for this measure.
